# Selenium, Stroke, and Infection: A Threefold Relationship; Where Do We Stand and Where Do We Go?

**DOI:** 10.3390/nu15061405

**Published:** 2023-03-15

**Authors:** Andreas Liampas, Panagiotis Zis, Georgios Hadjigeorgiou, George D. Vavougios

**Affiliations:** 1 Department of Neurology, Nicosia New General Hospital, Nicosia 2029, Cyprus; up1090698@upatras.gr; 2 Second Department of Neurology, School of Medicine, ‘Attikon’ University Hospital, National and Kapodistrian University of Athens, 124 62 Athens, Greece; 3 Medical School, University of Cyprus, Nicosia 2024, Cyprus

**Keywords:** selenium, coagulation, ischemic stroke, infection

## Abstract

Stroke is currently the second most common cause of death worldwide and a major cause of serious long-term morbidity. Selenium is a trace element with pleotropic effects on human health. Selenium deficiency has been associated with a prothrombotic state and poor immune response, particularly during infection. Our aim was to synthesize current evidence on the tripartite interrelationship between selenium levels, stroke, and infection. Although evidence is contradictory, most studies support the association between lower serum selenium levels and stroke risk and outcomes. Conversely, limited evidence on the role of selenium supplementation in stroke indicates a potentially beneficial effect of selenium. Notably, the relationship between stroke risk and selenium levels is bimodal rather than linear, with higher levels of serum selenium linked to disturbances of glucose metabolism and high blood pressure, morbidities which are, in turn, substrates for stroke. Another such substrate is an infection, albeit forming a bidirectional relationship with both stroke and the consequences of impaired selenium metabolism. Perturbed selenium homeostasis leads to impaired immune fitness and antioxidant capacity, which both favor infection and inflammation; specific pathogens may also contend with the host for transcriptional control of the selenoproteome, adding a feed-forward loop to this described process. Broader consequences of infection such as endothelial dysfunction, hypercoagulation, and emergent cardiac dysfunction both provide stroke substrates and further feed-forward feedback to the consequences of deficient selenium metabolism. In this review, we provide a synthesis and interpretation of these outlined complex interrelationships that link selenium, stroke, and infection and attempt to decipher their potential impact on human health and disease. Selenium and the unique properties of its proteome could provide both biomarkers and treatment options in patients with stroke, infection, or both.

## 1. Background

Stroke is the second most common cause of death worldwide (11.8% of all deaths) and a major cause of serious long-term morbidity [1,2]. Ischemic stroke (IS) is the most common type of stroke, comprising about 80% of the total cerebrovascular events [3]. Despite the decline of stroke mortality over the years, the number of IS-related deaths and morbidities and overall disability-adjusted life years (DALY) lost remains of great importance and increases in the course of time [4]. Although most risk factors are modifiable, including hypertension, diabetes mellitus, hyperlipidemia, and smoking [5] other non-modifiable variables such as age, sex, and genetics have also been considered as risk factors for stroke [6,7,8]. Studies on nutritional factors affecting stroke risk and outcomes have been generally focused on dyslipidemia, with micronutrients and trace elements being generally underexplored in the literature. 

Micronutrients or trace elements refer to nutritional factors required in specific, minute quantities by organisms and affect specific aspects of their physiological functions. One such micronutrient is selenium (Se) [9]. Selenium exerts multiple pleiotropic homeostatic roles on human health, several of which are interwoven with stroke etiopathogenesis and its outcomes, as well as infection. This tripartite relationship is not merely observational; many of the shared pathophysiological substrates that link infection and stroke find common grounds in Se biology. 

A central case in point for this concept arises from the observed interrelationships between serum Se concentration, the systemic inflammatory response, and multi-organ failure in sepsis patients. As selenoproteins are known to mitigate oxidative stress, coagulation, and immune fitness, these associations potentially reflect homeostatic adaptations aimed at preserving tissues and organs affected by the stress represented by sepsis [10]. Another example of the pertinence of Se biology arises from host–virus interactions, where host Se biology is actively contested by viruses in order to favor egress, with oxidative stress and hypercoagulable states representing secondary consequences. Moreover, selenium depletion secondary to infection may build up towards a prothrombotic state and impaired immune fitness, a milieu that has been shown to favor viral genomic instability potentially resulting in variants with greater pathogenicity [11]. 

The purpose of this critical review is to explore state-of-the-art evidence on the epidemiology, potential mechanisms, and possible interventions regarding selenium status, infection, and stroke. Furthermore, we aim to provide a concise report on current concepts regarding selenium supplementation in the specific setting of infectious disease and stroke, as well as their interplay.

### 1.1. Selenium Biology 

Selenium is a fundamental trace element of human biology and mediates its enzymic functions through selenoproteins [12]. Selenium is mainly found in soil and enters the food chain through plants or through external supplementation [9]. The daily selenium uptake differs from country to country and mostly depends on the soil content of selenium [13,14]. Apparently, there is no consensus on the appropriate daily intake of selenium required to achieve plateau concentrations of plasma glutathione peroxidase (GPx) [9]. The American Recommended Dietary Allowance (RDA), revised in April 2000, suggests 55 μg per day for both men and women, whereas in other countries the recommended doses are higher probably due to a lower selenium status of their population [15]. In the United Kingdom, (UK) the recommended dietary dose for an adult woman is 60 μg/day, whereas in lactating women and adult men the recommended dose is higher at 75 μg/day [16]. Plasma and serum selenium levels are commonly used for the measurement of selenium status, though selenoprotein P levels and glutathione peroxidase activity are also widely used [17,18].

Four forms of selenium are taken up and eventually incorporated into selenoproteins. Two sources can be found as organic compounds selenomethionine (SeMet) and selenocysteine (Sec), and two inorganic forms such as selenite and selenate [19]. The most prominent form of selenium ingested by humans is SeMet. Selenium is metabolized through the liver, where Selenoprotein P (SELENOP) is synthesized which transports selenium to other tissues and organs through the bloodstream [20]. The transported selenium is then converted to selenophosphate by intracellular selenium metabolic pathways. Eventually, selenium is excreted from the human body either through exhalation or via urine as small-molecule metabolites [21,22,23]. 

In humans, the selenium donor for selenocysteine (Sec) biosynthesis is selenophosphate, produced from selenite and the consumption of ATP in a reaction catalyzed by selenophosphate synthetase 2 (SPS2) [24].

Unlike other amino acids, Sec biosynthesis is uniquely dependent on its tRNA [25]. Initially, Sec tRNA undergoes aminoacetylation catalyzed by the seryl-tRNA synthetase 1 (SerRS) and formulates Ser t-RNASec. [26]. Subsequently, Ser-tRNASec is phosphorylated to form O-phosphoseryl-tRNASec (Sep-tRNASec) by Sep-tRNA kinase (PSTK) [24,27]. The third step requires the conversion of O-phosphoseryl-tRNA (Sec) to selenocysteinyl-tRNA (Sec) by the O-phosphoseryl-tRNA (Sec) selenium transferase (SEPSECS) [28]. 

Sec is encoded by the UGA codon, which would otherwise signal translation termination. Selenoprotein mRNAs contain the Sec insertion sequence (SECIS) element, a conserved steam and loop RNA structure beyond the UGA codon and within the 3′-UTR. SECIS is required for the recognition of selenocysteine UGA codons which is furthermore dependent on the elongation factor EFSec and the protein factor SECIS-binding protein 2 (SBP2). Following the delivery of the tRNA carrying selenocysteine to the UGA, the UGA codon is recognized as selenocysteine rather than a stop codon, and Sec is inserted into the elongating chain [29]. It should be noted that the SBP2′s redox state, governed by thioredoxin and glutaredoxin systems, is linked to its functions, indicating a regulatory feedback loop between oxidative stress buffering and selenoprotein translational rhythms [30,31].

The regulation of selenium metabolism and the selenoproteome is crucial for the appropriate function of several systems of human biology including the central nervous system, the endocrine system, muscle function, the cardiovascular system, and immunity [32,33]. Selenium abundance regulates redox homeostasis in a level-dependent manner, described as the selenium “paradox”, i.e., where its accumulation after a threshold leads to oxidative states [34]; this, however, does not refer to a true paradox despite its accounts in the literature, but rather the on bimodal effects dependent on depletion or abundance. 

In the following sections, we will review canonical roles for the selenoproteome that are actively implicated in infection and cerebrovascular disease.

### 1.2. Functional Classification of the Selenoproteome

As described previously, selenium is incorporated in the active form of the 21st amino acid selenocysteine (Sec), which is encoded by a UGA stop codon [31]. 

Its biological effects are exerted via both the selenoproteome and Se’s bioactive metabolites such as hydrogen selenide and methylated selenium compounds such as methylseleninic acid [33,35]. The latter molecules take part in biological processes such as DNA repair and epigenetics through redox reactions, with the selenoproteome in general representing a wide array of antioxidant proteins ubiquitous to the human body.

To date, the selenoproteome refers to the products of 25 selenoprotein-coding genes in humans; out of 25 selenoproteins, 13 of them have known functions [36], further classified into six subgroups; (i) peroxidase and reductase activities, (ii) hormone metabolism, (iii) protein folding, (iv) redox signaling, (v) Sec synthesis, and (vi) selenium transport [37,38]. Structurally, SELENOP is distinguished from the rest of the selenoproteins, since it contains multiple selenol amino acids per polypeptide, whereas all other characterized selenoproteins contain only one residue [39]. The gene *SELENOP*, which encodes the protein of the same name, is located on chromosome 5q31.1 [40]. The true functional repertoire stemming from selenoprotein coding genes may be more expansive, as alternative splicing and other post-translational mechanisms may produce subcellular specific isoforms [41] or create protein variants with novel functionalities [42].

Conversely, mutations in selenoproteins genes have varying consequences, as described above, stemming, however, from the resulting dysregulation of redox homeostasis. These mutations affect high-energy tissues such as muscle, the thyroid, and the brain, where tightly regulated mitigation of oxidative stress is essential for their function [43]. Studies provide strong evidence of a link between genetic variants in selenoproteins genes and risk for various chronic diseases [44]. Several single nucleotide polymorphisms (SNPs) that affect the selenoproteome and are associated with the disease have been identified; affected genes include *SELENOP*, *SEPHS1*, *GPX1*, *GPX3*, *GPX4*, *TXNRD1*, *TXNRD2*, *SELENOF*, *DIO2*, and *SELENOS* among others [44,45]. In particular, prostate cancer has been linked to variants *TXNRD1* and *TXNRD2* [46], colorectal cancer has been linked to variants *GPX1*, *GPX4*, *SELENOP*, *SELENOF*, and *TXNRD1* [47,48,49,50], lung cancer has been linked to variants *GPX1*, *GPX4*, and *SELENOF* [51,52,53] and increased risk of cardiovascular disease has been linked to variants *GPX1* and *SELENOS* [54,55]. 

A general principle for selenoproteins is that they take up homeostatic roles in multiple tissues by coupling metabolic and other cellular housekeeping functions with redox homeostasis, a modus operandi reflected both in health and disease [56]. In the subsequent sections, we intend to explore the role of selenium and the selenoproteome in conditions that represent substrates and shape the course of stroke and infection, namely immunity and coagulation.

### 1.3. The Selenoproteome and Immunity

The selenoproteome plays a fundamental role in the regulation of diverse aspects of immunity [57] and mitigates the prooxidant milieu developed during inflammation [34,58]. The role of selenium in immunity appears to be bidirectional, capable of both enhancing and mitigating inflammation secondary to infection. These bimodal effects indicate an operational buffer zone for Se levels, beyond which both deficiency and excess can be harmful.

In particular, selenium depletion and decreased selenoprotein expression have been associated with higher levels of inflammatory cytokines in a variety of tissues such as the gastrointestinal tract, the uterus, mammary gland tissues, and others [33]. However, increased selenium intake may lead to a setting-specific upregulation of inflammatory processes. For example, it can lead to the upregulation of stress-responsive selenoproteins implicated in inflammation and interferon γ (IFNγ) responses [59]. Moreover, it has been demonstrated that a diet rich in selenium is associated with potentially increased activation of leukocytes [60], proliferation and differentiation of immune cells, and increased production of IFNγ and other cytokines [61,62]. Selenium studies on the neutrophil-to-lymphocyte ratio (NLR), an index of systemic inflammation [61,62,63,64], have also indicated interrelationships with Se homeostasis. Selenium and other micronutrients have been negatively correlated with NRL levels in patients with COVID-19 [65], indicating the protective role of selenium supplementation in severe inflammatory conditions.

Acquired selenium deficiency may lead to immune dysregulation [33], albeit with differential, abundance-dependent effects on different aspects of immunity; specifically, adequate selenium intake and homeostasis have been shown to be instrumental in maintaining immune surveillance and the capacity to mobilize and regulate immune mechanisms. Conversely, Se dyshomeostasis has been linked with perturbed innate and humoral immune responses [66]. Despite the pivotal role of selenium in many immune cell functions, data regarding selenium supplementation as an approach to boost immunity in the general population are limited.

### 1.4. Selenium and Coagulation

Aside from immunity, selenium is critical for the maintenance of adequate coagulation mechanisms. GPx activity and the modulation of lipid hydroperoxides levels. Se plays a critical role in the arachidonic acid (AA) cascade [67]. During the AA metabolism, GPx acts as a scavenger of hydrogen peroxide and furthermore inhibits the production of both thromboxane A2 (TXA2) and lipoxygenase products [68,69]. 

In particular, GPx-3, a member of the selenocysteine-containing GPx family is a major antioxidant enzyme and is the only one found in the extracellular space. Deficiency of this enzyme is associated with a prothrombotic state and vascular dysfunction [70]. GPx furthermore regulates the bioavailability of nitric oxide (NO), which has a potential inhibitory effect on platelet activation and aggregation [71]; conversely, its abrogation would therefore contribute to potential hyperactivity of otherwise normal functioning platelets, culminating in a prothrombotic state. Selenium deficiency as an initial step has been shown to contribute to and enhance the prothrombotic state in a variety of medical conditions including severe sepsis or septic shock [72,73] and cancer [74]. There is an inverse association of selenium levels and oxidative stress in patients with sepsis or cancer survivors as well as those associated with severe trauma [75], which in turn may predispose one to a hypercoagulable state by impairing red blood cell functions, promoting to endothelial dysfunction, and, finally, activating platelets and leukocytes—events that collaboratively and independently affect an imbalance of the regulatory aspects of hemostasis [76].

## 2. The Role of Selenium on Cerebrovascular Risk

In the previous sections, we expanded on the role of selenium on factors that may increase the susceptibility to cerebrovascular events. Aside from these, there are several studies that have attempted to explore the direct role of selenium in stroke. 

In an observational cross-sectional study (N = 12,095), authors found an inverse cross-sectional association between low levels of total selenium and prevalence of stroke mostly in specific population subgroups; male, elderly, white, and people with higher education and higher income [77]. In a case-control study of Canadian Inuit who generally display high plasma Se concentrations, authors found that blood selenium (geometric mean: 260 μg/L vs. 319 μg/L) and dietary selenium (144 μg/day vs. 190 μg/day) were lower in patients with stroke compared to participants without stroke, in whom selenium exposure was higher [78]. Hu et al. found that each 50-μg/L increase in blood selenium and in dietary selenium led to a 38% and 30% reduction in the prevalence of stroke, respectively. The inverse association was also supported in a case-control study with more than 1000 Chinese subjects, where Wen et al. demonstrated the potential protective role of selenium in cardiovascular disease, since lower levels of blood selenium were linked to a higher risk of ischemic stroke [79]. The protective role of selenium on ischemic stroke was also observed by Hu et al. in a case-control study, but the negative association was demonstrated only in males [80]. 

In a meta-analysis of 12 observational studies, authors yielded a negative association between selenium levels and stroke [81], though the subgroup analysis did not confirm this association in the prospective cohort group. In a case-control study, Mironczuk et al. demonstrated higher Cu/Se and Cu/Zn molar ratios in patients with cardio-embolic stroke, but relatively low Cu/Se molar ratios in patients with small vessel disease [82].

Fang et al. conducted a mendelian randomization analysis, where four SNPs (rs921943, rs6859667, rs6586282, and rs1789953) significantly associated with selenium levels were studied [83]. However, there is insufficient evidence to support a causal link between genetically predicted selenium levels and ischemic stroke and its subtypes were demonstrated. Similarly, in a cohort study (*n* = 1103) Wei et al. failed to demonstrate an association between selenium concentrations and cerebrovascular events in a 15-year follow-up period [84]. In a longitudinal cohort study (*n* = 27,770), authors studied the role of trace elements in the environment on stroke and found that higher levels of environmental selenium exposure were associated with a higher risk of stroke [85]. Indirect associations between stroke risk factors and selenium levels stem also from a limited number of studies with somewhat conflicting results. Wu et al. conducted a Mendelian randomization analysis and demonstrated a causal effect of selenium levels on beneficial lipid profile, including decreased total cholesterol (TC) and low-density lipoprotein cholesterol (LDL-C) and glucose profile, including HbA1c levels and insulin indicating a protective role of selenium on cardiovascular events [86]; however, there is a caution on that evidence since levels of selenium above the physiological range could be harmful on the glucose metabolism [87,88].

Current literature provides limited evidence on the role of selenium supplementation during the acute phase of stroke. Two randomized placebo-controlled trials (RCTs) evaluated the effect of intravenous administration of sodium selenite pentahydrate (Selenase) on short-term and long-term acute ischemic stroke outcomes [89,90]. Both studies demonstrated that selenium supplementation improved short-term outcomes in acute stroke patients in the context of neurological deficits and degree of disability. Additionally, Ramezani et al. showed increased activity of antioxidant enzymes in patients who received Selenase, whereas serum inflammatory markers were lower in the Selenase group (N = 25) compared to the placebo group (N = 25) [90]. However, both studies failed to yield a beneficial effect of selenium supplementation during the acute phase of stroke in the context of long-term outcomes. Furthermore, it should also be noted that these studies indicate that the role of selenium supplementation on the outcome of cerebrovascular events is to date contradictory, in part due to the heterogeneity between study design, populations included, and measurement methods [77,78,79,80,81,82,83,84,85,86,87].

## 3. Selenium, Stroke, and Infection: From Clinical Observations to Molecular Mechanisms 

The relationship between stroke and infection is bidirectional: stroke increases the predisposition to infections, and infections represent a risk factor for stroke [91]. During infection, hypercoagulable states represent a broad response intended to limit pathogen spread [92]. For instance, inflammatory cytokines promote hypercoagulation diffusely and locally as a ‘walling off’ effect. Infection and specific pathogens may be associated with persistent prothrombotic states, reflecting lasting epigenetic sequelae [92]. The interplay between sepsis and hemostasis has been shown to implicate selenium status in critically ill patients, with lower levels affecting reflecting adverse outcomes [10]. Conversely, a meta-analysis of supplementation trials in similar patients has shown an ameliorating effect on total mortality [93]. A broad concept for the underlying mechanism may be micronutrient depletion, as cells mobilize antioxidants such as GPx to survive both their own hypermetabolic states and the pro-oxidant milieu [94]. In a murine stroke model, adequate GPx scavenging limited hypoxic and ischemia-reperfusion injury [95]; this model may be applicable to stroke patients, where a reduction of glutathione peroxidase but not other selenoproteins is noted [96]. Within this context, a prior or concomitant infection could lead to selenium depletion, a concomitant depletion of the selenium-dependent glutathione peroxidase, and the abrogation of their neuroprotection. In the absence of infection, the same chain of events could be instigated by chronically low dietary intake. 

It should be noted viruses may not only capitalize on a vulnerable state established in the setting of selenium dyshomeostasis, but also actively target the host selenoproteome as part of their lifecycles. Specifically, the selenoproteome is targeted by the molecular armamentarium deployed by viruses such as HIV- 1 and EBOV in a bid to gain transcriptional control of selenoprotein synthesis. The procedures via which this is achieved are unique host–virus interactions such as antisense tethering (ATI) [97]. ATIs between viral and selenoproteome mRNAs reflect a gain-of-function procedure during the viral lifecycle, using the captured host selenocysteine insertion sequence (SECIS) element to bypass a UGA stop codon and read through it as selenocysteine [97,98]. Virus–host ATIs utilize an established readthrough mechanism that governs the incorporation of selenocysteine in selenoproteins albeit with contextual UGA to selenocysteine efficiency [99], potentially intended as a limiting step in their translational rhythms [100]. The bioavailability and utilization of other metal ions involved in cellular antiviral defense also impact selenium trafficking. Intracellular copper trafficking, utilized to saturate phagosomes and in turn modulated by pathogens themselves [101], directly impacts selenium biology. The specific mechanisms involved implicate the disruption of UGA recoding by copper, downregulating selenoproteome expression [102]. Taken together, these findings indicate that Se homeostasis is targeted at a molecular level via a broad spectrum of pathogens; in turn, both host defense and selenoprotein dysfunction feedback to hypercoagulable states which would be further exacerbated by the eventual depletion of selenium levels.

## 4. Discussion

In this narrative review, we explored the robust tripartite relationship between selenium levels, stroke, and infection. 

Collectively, the studies presented herein point towards two major mechanisms that may account for the threefold relationship between selenium biology, stroke, and infection. The first may lie in selenium’s role in antioxidant cascades and its specific function cardioprotective agent displayed both against non-organic compounds [103,104,105] and bacterial toxins [106]. Oxidative stress plays a significant role in the evolution and by extent, systemic and tissue-specific consequences of stroke [107] and infection [108]. Selenium’s role in the mitigation of oxidative stress is mediated via its incorporation in the selenoproteome, an antioxidant proteome that includes a key molecule, glutathione. Homeostatically, these enzymes ameliorate stress and promote cellular survival both in the setting of basal conditions (i.e., oxidative phosphorylation) and during specific insults. As an example, during the acute phase of ischemic stroke, interlinked oxidative sensing and cytoprotective pathways activate due to the hypoxic states and upregulate the selenoproteome pathway [109,110,111]. As a case in point, glutathione peroxidase, major selenoproteins, and antioxidants have been shown to confer further specific effects in reducing stroke volume and ischemic injury [112]; in the setting of bacterial infection, glutathione ameliorates tissue level oxidative stress [113] and modulates the survival of immune cells [114]. Collectively, the maintenance of selenium homeostasis represents an important pathway of cellular survival that when compromised abrogates major cytoprotective mechanisms that function against multiple stressors—which, in the case of stroke and infection, may even synergize.

From a physiological perspective, selenium uptake may represent a primary modifiable factor that may account for differential susceptibility to stroke and infection. The optimal concentration levels in adult human blood serum are 80 to 120 mcg/L, though there is a great variety of normal values from country to country [14,115]. However, selenium concentration is age dependent [116,117]. Because of age-related nutritional adaptations, children require less circulating selenium than adults [116,118]. Aside from age, there may be a biological basis for gender differences and selenium metabolism. Animal studies in murine models investigated the association between estrogen and selenium status and demonstrated that estrogen status affects Se metabolism by modulating SELENOP, and to an extent Se transport [119]. Moreover, absorption of selenocompounds by the intestinal epithelia seems to be greater in females (~96%) [120] compared to males (~76%) [121]. A recent study in western Romania found that selenium uptake was lower in females compared to males [115]. Although these studies cannot provide a mechanism for these differences, they nevertheless should be taken into account when interpreting data and designing future studies. On the same premise, it should be noted that measuring the precise dietary selenium intake may be difficult [14], considering food processing such as cooking which could produce a loss of up to 40% by volatilization [118,122]. One such proposed technique is the geometric mean evaluation of whole blood and urinary selenium levels using sequential measurements [123].

**Figure 1:**
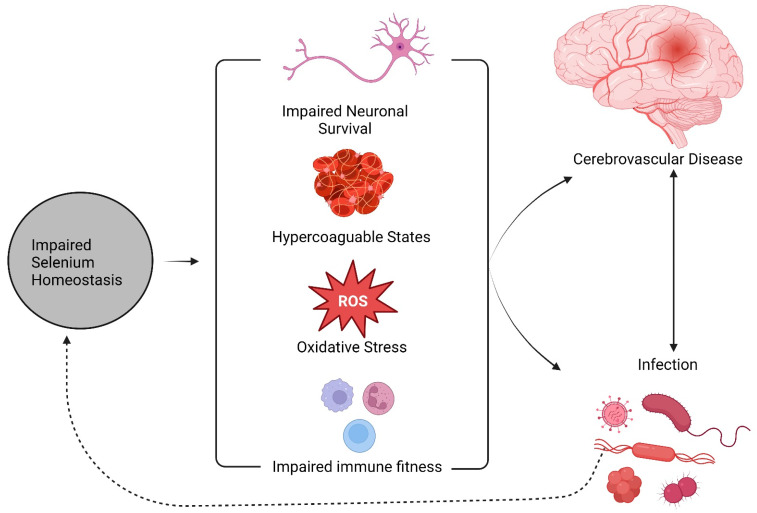
The tripartite relationship between impaired selenium homeostasis, stroke, and infection. The consequences of impaired selenium homeostasis help establish a milieu of multiple failing cellular functions that mitigate oxidative stress, regulate immune fitness, maintain coagulation, and via a combination of the above, promote neuronal survival. Once established, these major dysfunctions present primers for infection and stroke. As an example, impaired selenium homeostasis would establish a pro-oxidant milieu that could furthermore dysregulate coagulation and abrogate neurons of their means to resist hypoxia. In this setting, cerebrovascular disease could develop, and once established, greatly impact the affected tissue. In a similar manner, defunct immune surveillance secondary to impaired immune dysregulation would effectively reflect increased susceptibility to pathogens that would otherwise be successfully restricted; furthermore, even in the setting of the eventual mobilization of immune responses, depleted selenium would also abrogate the fine-tuning of the ongoing inflammation. It should also be noted that the mechanisms at play here are interconnected and may form feed-forward loops on the initial events presented herein. As such, impaired immune surveillance secondary to selenium depletion could further be exacerbated by infection, i.e., viruses that can modulate immune responses and target selenium metabolism in turn. Sepsis is another such example where depletion of antioxidant selenoproteins, oxidative stress, and hypercoagulable states could build up to cerebrovascular disease. In this figure, a select rather than an exhaustive number of salient features and potential feed-forward loops secondary to impaired selenium homeostasis is presented; the feedback loop between infection and impaired selenium homeostasis is shown with a discontinuous arrow, whereas the interrelationships between each individual consequence are implied by the inclusion in brackets. The figure was created with BioRender.com (Available from: https://app.biorender.com; Accessed 13 December 2022).

From a pathophysiological perspective, disruptions of Se homeostasis occur both as a substrate and a consequence of infection and stroke—two conditions uniquely intertwined (Figure 1). As we have previously mentioned, the association between stroke and infection is bidirectional. Several infections or vaccinations have been shown to contribute to an increased risk of stroke [124,125,126,127] and particularly patients with sepsis and urinary infection are at greater risk [127,128,129] even 1 year after sepsis hospitalization [128]. Many pathogenic mechanisms have been proposed such as direct invasion of the vascular endothelial wall by pathogens and the instigation of focal inflammation, acceleration of atherosclerosis through induction of proinflammatory cytokines (such as TNF-alpha, interleukin 2) in response to specific antigenic stimuli, infection-induced cardiac arrhythmias and infectious burden due to chronic infections which may dysregulate the magnitude of immune responses [130]. Although infection can lead to stroke, stroke also induces impairment of immunological competence which increases the risk of infection [91]. Moreover, immobilization due to motor paralysis or dysphagia as post-stroke sequelae suggests a risk of aspiration pneumonia and further opportunistic infections [131].

While the mechanistic evidence on the importance of selenium homeostasis converges, real-world data on the potential implementation of selenium supplementation are not consistent and are provided by a heterogeneous body of studies. To date, there is significant controversy regarding the association between measured selenium levels and cerebrovascular risk, with most epidemiological studies suggesting an inverse cross-sectional association. Similarly, low selenium serum levels have been associated with increased cardiovascular events and mortality [117,132,133], yielding the anticoagulant role of selenium through modulation of GPx activity [134]. However, levels of selenium above the normal range have been associated with dysregulated glucose metabolism [87] and hypertension [135], which are both individually synergizing risk factors for cerebrovascular and cardiovascular events; this may indicate that measured selenium levels are linearly correlated with vascular changes, with higher measured levels reflecting dysregulation rather than abundance. In particular, total selenium intake above 300 μg/d and supplements and high doses of selenium are considered harmful and should be avoided. Evidence regarding the effect of selenium administration during the acute phase of stroke is limited, though promising results were demonstrated in two small RCTs [89,90].

Our results should be interpreted with due caution given the limitations of our study design. Firstly, this is a narrative review, hence our article does not meet specific criteria to help mitigate bias as they lack explicit criteria for article selection. Secondly, our search is limited to publications in PubMed; therefore, it is possible that studies that were solely indexed in other databases were missed. Moreover, there was a great deal of heterogeneity in terms of the administration of Se supplementation. Collectively, these studies on real-world implementation of Se supplementation indicate major gaps in knowledge and inconsistent reports on how supplements affect health. As Se confers many of its actions via incorporation to selenoproteins, bioavailability, as measured by various techniques, may not be indicative of biological activity, and by extension may not be able to reflect whether a beneficial or detrimental effect would be conferred [136]. By contrast, measurements of antioxidant capacity conferred by selenoproteins such as glutathione peroxidase perform more consistently in the literature [137,138]. These points indicate that Se supplementation and its use as a biomarker would require a paradigm shift that recognizes the complexity of its biology and the limitations of current methods. Furthermore, data from the studies presented herein indicate that supplementation strategies should also take into account additional risk-to-benefit analyses, as well as account for the specific setting in which such supplementation could be harmful rather than beneficial.

## 5. Conclusions

In this work, we review a growing body of literature that outlines the relationship between selenium biology, stroke, and infection. Several lines of evidence suggest that lower selenium levels are associated with worse stroke outcomes. Future studies should consider stroke subtypes and comorbidities in evaluating serum Se levels and Se supplementation. In the setting of infections and sepsis, and specifically those due to pathogens that are known to install hypercoagulable states, e.g., COVID-19, Se's role is still not adequately explored but may provide an accessible biomarker and potential treatment. In a broader context, interventions focusing on adequate selenium uptake could ameliorate this risk, whereas the selenium supplementation, aiming to establish or maintain levels within the normal range following stroke, should further be explored as a neuroprotective strategy limiting stroke recurrence through larger RCTs. Future research should address the current limitations of Se measurements and the importance of its incorporation in the selenoproteome, potentially aiming to develop better assays and therapeutics. Furthermore, current concepts in Se supplementation should be informed of the potential risks of supplementation, and provide a specific outline of patient groups like to benefit, and conversely, likely to experience adverse events.

## Data Availability

Not applicable.

## References

[1] Global, Regional, and National Life Expectancy, All-Cause Mortality, and Cause-Specific Mortality for 249 Causes of Death, 1980–2015: A Systematic Analysis for the Global Burden of Disease Study 2015—The Lancet. https://www.thelancet.com/journals/lancet/article/PIIS0140-6736(16)31012-1/fulltext.

[2] Campbell B.C.V., De Silva D.A., Macleod M.R., Coutts S.B., Schwamm L.H., Davis S.M., Donnan G.A. (2019). Ischaemic Stroke. Nat. Rev. Dis. Primer.

[3] Goldstein L.B., Adams R., Becker K., Furberg C.D., Gorelick P.B., Hademenos G., Hill M., Howard G., Howard V.J., Jacobs B. (2001). Primary Prevention of Ischemic Stroke: A Statement for Healthcare Professionals from the Stroke Council of the American Heart Association. Stroke.

[4] Krishnamurthi R.V., Feigin V.L., Forouzanfar M.H., Mensah G.A., Connor M., Bennett D.A., Moran A.E., Sacco R.L., Anderson L.M., Truelsen T. (2013). Global Burden of Diseases, Injuries, Risk Factors Study 2010 (GBD 2010); GBD Stroke Experts Group. Global and Regional Burden of First-Ever Ischaemic and Haemorrhagic Stroke during 1990-2010: Findings from the Global Burden of Disease Study 2010. Lancet Glob. Health.

[5] Feigin V.L., Roth G.A., Naghavi M., Parmar P., Krishnamurthi R., Chugh S., Mensah G.A., Norrving B., Shiue I., Ng M. (2016). Global Burden of Diseases, Injuries and Risk Factors Study 2013 and Stroke Experts Writing Group. Global Burden of Stroke and Risk Factors in 188 Countries, during 1990–2013: A Systematic Analysis for the Global Burden of Disease Study 2013. Lancet Neurol..

[6] Xian Y., Holloway R.G., Smith E.E., Schwamm L.H., Reeves M.J., Bhatt D.L., Schulte P.J., Cox M., Olson D.M., Hernandez A.F. (2014). Racial/Ethnic Differences in Process of Care and Outcomes among Patients Hospitalized with Intracerebral Hemorrhage. Stroke.

[7] Owolabi M., Sarfo F., Howard V.J., Irvin M.R., Gebregziabher M., Akinyemi R., Bennett A., Armstrong K., Tiwari H.K., Akpalu A. (2017). SIREN-REGARDS Collaboration (Stroke Investigative Research and Educational Network–Reasons for Geographic and Racial Differences in Stroke). Stroke in Indigenous Africans, African Americans, and European Americans: Interplay of Racial and Geographic Factors. Stroke.

[8] Katan M., Luft A. (2018). Global Burden of Stroke. Semin. Neurol..

[9] Rayman M.P. (2000). The Importance of selenium to Human Health. Lancet Lond. Engl..

[10] Sakr Y., Reinhart K., Bloos F., Marx G., Russwurm S., Bauer M., Brunkhorst F. (2007). Time Course and Relationship between Plasma selenium Concentrations, Systemic Inflammatory Response, Sepsis, and Multiorgan Failure. Br. J. Anaesth..

[11] Martinez S.S., Huang Y., Acuna L., Laverde E., Trujillo D., Barbieri M.A., Tamargo J., Campa A., Baum M.K. (2021). Role of selenium in Viral Infections with a Major Focus on SARS-CoV-2. Int. J. Mol. Sci..

[12] Hogan C., Perkins A.V. (2022). Selenoproteins in the Human Placenta: How Essential Is selenium to a Healthy Start to Life?. Nutrients.

[13] Reilly C. (1996). Selenium in Food and Health.

[14] Stoffaneller R., Morse N.L. (2015). A Review of Dietary selenium Intake and selenium Status in Europe and the Middle East. Nutrients.

[15] Zeece M., Zeece M. (2020). Chapter Five—Vitamins and Minerals. Introduction to the Chemistry of Food.

[16] (1991). Dietary Reference Values for Food Energy and Nutrients for the United Kingdom. Report of the Panel on Dietary Reference Values of the Committee on Medical Aspects of Food Policy. Rep. Health Soc. Subj..

[17] Ross A.C., Caballero B.H., Cousins R.J., Tucker K.L., Ziegler T.R. (2012). Modern Nutrition in Health and Disease.

[18] Ashton K., Hooper L., Harvey L.J., Hurst R., Casgrain A., Fairweather-Tait S.J. (2009). Methods of Assessment of selenium Status in Humans: A Systematic Review. Am. J. Clin. Nutr..

[19] Kang D., Lee J., Wu C., Guo X., Lee B.J., Chun J.-S., Kim J.-H. (2020). The Role of selenium Metabolism and Selenoproteins in Cartilage Homeostasis and Arthropathies. Exp. Mol. Med..

[20] Hill K.E., Zhou J., McMahan W.J., Motley A.K., Atkins J.F., Gesteland R.F., Burk R.F. (2003). Deletion of Selenoprotein P Alters Distribution of selenium in the Mouse. J. Biol. Chem..

[21] McConnell K.P., Portman O.W. (1952). Excretion of Dimethyl Selenide by the Rat. J. Biol. Chem..

[22] Byard J.L. (1969). Trimethyl Selenide. A Urinary Metabolite of Selenite. Arch. Biochem. Biophys..

[23] Hadrup N., Ravn-Haren G. (2021). Absorption, Distribution, Metabolism and Excretion (ADME) of Oral selenium from Organic and Inorganic Sources: A Review. J. Trace Elem. Med. Biol. Organ Soc. Miner. Trace Elem. GMS.

[24] Xu X.-M., Carlson B.A., Irons R., Mix H., Zhong N., Gladyshev V.N., Hatfield D.L. (2007). Selenophosphate Synthetase 2 Is Essential for Selenoprotein Biosynthesis. Biochem. J..

[25] Lee B.J., Worland P.J., Davis J.N., Stadtman T.C., Hatfield D.L. (1989). Identification of a Selenocysteyl-TRNA(Ser) in Mammalian Cells That Recognizes the Nonsense Codon, UGA. J. Biol. Chem..

[26] Holman K.M., Puppala A.K., Lee J.W., Lee H., Simonović M. (2017). Insights into Substrate Promiscuity of Human Seryl-TRNA Synthetase. RNA N. Y. N.

[27] Carlson B.A., Xu X.-M., Kryukov G.V., Rao M., Berry M.J., Gladyshev V.N., Hatfield D.L. (2004). Identification and Characterization of Phosphoseryl-TRNA[Ser]Sec Kinase. Proc. Natl. Acad. Sci. USA.

[28] Palioura S., Herkel J., Simonović M., Lohse A.W., Söll D. (2010). Human SepSecS or SLA/LP: Selenocysteine Formation and Autoimmune Hepatitis. Biol. Chem..

[29] Hoffman K.S., Crnković A., Söll D. (2018). Versatility of Synthetic TRNAs in Genetic Code Expansion. Genes.

[30] Papp L.V., Lu J., Striebel F., Kennedy D., Holmgren A., Khanna K.K. (2006). The Redox State of SECIS Binding Protein 2 Controls Its Localization and Selenocysteine Incorporation Function. Mol. Cell. Biol..

[31] Shetty S.P., Shah R., Copeland P.R. (2014). Regulation of Selenocysteine Incorporation into the selenium Transport Protein, Selenoprotein P. J. Biol. Chem..

[32] Rayman M.P. (2012). Selenium and Human Health. Lancet Lond. Engl..

[33] Avery J.C., Hoffmann P.R. (2018). selenium, Selenoproteins, and Immunity. Nutrients.

[34] Kyung L., Daewon J. (2012). Bimodal Actions of selenium Essential for Antioxidant and Toxic Pro-Oxidant Activities: The selenium Paradox (Review). Mol. Med. Rep..

[35] Ip C., Thompson H.J., Zhu Z., Ganther H.E. (2000). In Vitro and in Vivo Studies of Methylseleninic Acid: Evidence That a Monomethylated selenium Metabolite Is Critical for Cancer Chemoprevention. Cancer Res..

[36] Kryukov G.V., Castellano S., Novoselov S.V., Lobanov A.V., Zehtab O., Guigó R., Gladyshev V.N. (2003). Characterization of Mammalian Selenoproteomes. Science.

[37] Papp L.V., Lu J., Holmgren A., Khanna K.K. (2007). From selenium to Selenoproteins: Synthesis, Identity, and Their Role in Human Health. Antioxid. Redox Signal..

[38] Reeves M.A., Hoffmann P.R. (2009). The Human Selenoproteome: Recent Insights into Functions and Regulation. Cell. Mol. Life Sci. CMLS.

[39] Arteel G.E., Klotz L.-O., Buchczyk D.P., Sies H., Sies H., Packer L. (2002). Selenoprotein P. Protein Sensors and Reactive Oxygen Species—Part A: Selenoproteins and Thioredoxin. Methods in Enzymology.

[40] Hill K.E., Dasouki M., Phillips J.A., Burk R.F. (1996). Human Selenoprotein P Gene Maps to 5q31. Genomics.

[41] Labunskyy V.M., Hatfield D.L., Gladyshev V.N. (2014). Selenoproteins: Molecular Pathways and Physiological Roles. Physiol. Rev..

[42] Damdimopoulos A.E., Miranda-Vizuete A., Treuter E., Gustafsson J.-A., Spyrou G. (2004). An Alternative Splicing Variant of the Selenoprotein Thioredoxin Reductase Is a Modulator of Estrogen Signaling. J. Biol. Chem..

[43] Santesmasses D., Gladyshev V.N. (2021). Pathogenic Variants in Selenoproteins and Selenocysteine Biosynthesis Machinery. Int. J. Mol. Sci..

[44] Méplan C. (2015). selenium and Chronic Diseases: A Nutritional Genomics Perspective. Nutrients.

[45] Gladyshev V.N., Arnér E.S., Berry M.J., Brigelius-Flohé R., Bruford E.A., Burk R.F., Carlson B.A., Castellano S., Chavatte L., Conrad M. (2016). Selenoprotein Gene Nomenclature. J. Biol. Chem..

[46] Méplan C., Rohrmann S., Steinbrecher A., Schomburg L., Jansen E., Linseisen J., Hesketh J. (2012). Polymorphisms in Thioredoxin Reductase and Selenoprotein K Genes and selenium Status Modulate Risk of Prostate Cancer. PLoS ONE.

[47] Peters U., Chatterjee N., Hayes R.B., Schoen R.E., Wang Y., Chanock S.J., Foster C.B. (2008). Variation in the Selenoenzyme Genes and Risk of Advanced Distal Colorectal Adenoma. Cancer Epidemiol. Biomark. Prev. Publ. Am. Assoc. Cancer Res. Cosponsored Am. Soc. Prev. Oncol..

[48] Méplan C., Hughes D.J., Pardini B., Naccarati A., Soucek P., Vodickova L., Hlavatá I., Vrána D., Vodicka P., Hesketh J.E. (2010). Genetic Variants in Selenoprotein Genes Increase Risk of Colorectal Cancer. Carcinogenesis.

[49] Polymorphisms in the Selenoprotein S and 15-kDa Selenoprotein Genes Are Associated with Altered Susceptibility to Colorectal Cancer—PubMed. https://pubmed.ncbi.nlm.nih.gov/21052528/.

[50] Mukhtar M., Ashfield N., Vodickova L., Vymetalkova V., Levy M., Liska V., Bruha J., Bendova P., O’Sullivan J., Doherty G. (2022). The Associations of Selenoprotein Genetic Variants with the Risks of Colorectal Adenoma and Colorectal Cancer: Case-Control Studies in Irish and Czech Populations. Nutrients.

[51] Yang P., Bamlet W.R., Ebbert J.O., Taylor W.R., de Andrade M. (2004). Glutathione Pathway Genes and Lung Cancer Risk in Young and Old Populations. Carcinogenesis.

[52] Jaworska K., Gupta S., Durda K., Muszyńska M., Sukiennicki G., Jaworowska E., Grodzki T., Sulikowski M., Waloszczyk P., Wójcik J. (2013). A Low selenium Level Is Associated with Lung and Laryngeal Cancers. PLoS ONE.

[53] Jablonska E., Gromadzinska J., Sobala W., Reszka E., Wasowicz W. (2008). Lung Cancer Risk Associated with selenium Status Is Modified in Smoking Individuals by Sep15 Polymorphism. Eur. J. Nutr..

[54] Hamanishi T., Furuta H., Kato H., Doi A., Tamai M., Shimomura H., Sakagashira S., Nishi M., Sasaki H., Sanke T. (2004). Functional Variants in the Glutathione Peroxidase-1 (GPx-1) Gene Are Associated with Increased Intima-Media Thickness of Carotid Arteries and Risk of Macrovascular Diseases in Japanese Type 2 Diabetic Patients. Diabetes.

[55] Cox A.J., Lehtinen A.B., Xu J., Langefeld C.D., Freedman B.I., Carr J.J., Bowden D.W. (2013). Polymorphisms in the Selenoprotein S Gene and Subclinical Cardiovascular Disease in the Diabetes Heart Study. Acta Diabetol..

[56] Uhlén M., Fagerberg L., Hallström B.M., Lindskog C., Oksvold P., Mardinoglu A., Sivertsson Å., Kampf C., Sjöstedt E., Asplund A. (2015). Proteomics. Tissue-Based Map of the Human Proteome. Science.

[57] Hoffmann P.R., Berry M.J. (2008). The Influence of selenium on Immune Responses. Mol. Nutr. Food Res..

[58] Huang Z., Rose A.H., Hoffmann P.R. (2012). The Role of selenium in Inflammation and Immunity: From Molecular Mechanisms to Therapeutic Opportunities. Antioxid. Redox Signal..

[59] Tsuji P.A., Carlson B.A., Anderson C.B., Seifried H.E., Hatfield D.L., Howard M.T. (2015). Dietary selenium Levels Affect Selenoprotein Expression and Support the Interferon-γ and IL-6 Immune Response Pathways in Mice. Nutrients.

[60] Bentley-Hewitt K.L., Chen R.K.-Y., Lill R.E., Hedderley D.I., Herath T.D., Matich A.J., McKenzie M.J. (2014). Consumption of selenium-Enriched Broccoli Increases Cytokine Production in Human Peripheral Blood Mononuclear Cells Stimulated Ex Vivo, a Preliminary Human Intervention Study. Mol. Nutr. Food Res..

[61] Buonacera A., Stancanelli B., Colaci M., Malatino L. (2022). Neutrophil to Lymphocyte Ratio: An Emerging Marker of the Relationships between the Immune System and Diseases. Int. J. Mol. Sci..

[62] Regolo M., Vaccaro M., Sorce A., Stancanelli B., Colaci M., Natoli G., Russo M., Alessandria I., Motta M., Santangelo N. (2022). Neutrophil-to-Lymphocyte Ratio (NLR) Is a Promising Predictor of Mortality and Admission to Intensive Care Unit of COVID-19 Patients. J. Clin. Med..

[63] Cataudella E., Giraffa C.M., Di Marca S., Pulvirenti A., Alaimo S., Pisano M., Terranova V., Corriere T., Ronsisvalle M.L., Di Quattro R. (2017). Neutrophil-To-Lymphocyte Ratio: An Emerging Marker Predicting Prognosis in Elderly Adults with Community-Acquired Pneumonia. J. Am. Geriatr. Soc..

[64] Corriere T., Di Marca S., Cataudella E., Pulvirenti A., Alaimo S., Stancanelli B., Malatino L. (2018). Neutrophil-to-Lymphocyte Ratio Is a Strong Predictor of Atherosclerotic Carotid Plaques in Older Adults. Nutr. Metab. Cardiovasc. Dis. NMCD.

[65] Zhou S., Zhang F., Chen F., Li P., He Y., Wu J., Dong L., Wang C., Wang X., Zhang W. (2022). Micronutrient Level Is Negatively Correlated with the Neutrophil-Lymphocyte Ratio in Patients with Severe COVID-19. Int. J. Clin. Pract..

[66] Razaghi A., Poorebrahim M., Sarhan D., Björnstedt M. (2021). selenium Stimulates the Antitumour Immunity: Insights to Future Research. Eur. J. Cancer Oxf. Engl. 1990.

[67] Eisenmann C.J., Miller R.K. (1995). The Effect of selenium Compounds (Selenite, Selenate, Ebselen) on the Production of Thromboxane and Prostacyclin by the Human Term Placenta in Vitro. Toxicol. Appl. Pharmacol..

[68] Perona G., Schiavon R., Guidi G.C., Veneri D., Minuz P. (1990). selenium Dependent Glutathione Peroxidase: A Physiological Regulatory System for Platelet Function. Thromb. Haemost..

[69] Irani K. (2000). Oxidant Signaling in Vascular Cell Growth, Death, and Survival: A Review of the Roles of Reactive Oxygen Species in Smooth Muscle and Endothelial Cell Mitogenic and Apoptotic Signaling. Circ. Res..

[70] Jin R.C., Mahoney C.E., Coleman Anderson L., Ottaviano F., Croce K., Leopold J.A., Zhang Y.-Y., Tang S.-S., Handy D.E., Loscalzo J. (2011). Glutathione Peroxidase-3 Deficiency Promotes Platelet-Dependent Thrombosis in Vivo. Circulation.

[71] Loscalzo J. (2001). Nitric Oxide Insufficiency, Platelet Activation, and Arterial Thrombosis. Circ. Res..

[72] Kong L., Wu Q., Liu B. (2021). The impact of selenium administration on severe sepsis or septic shock: A meta-analysis of randomized controlled trials. Afr. Health Sci..

[73] French L., Temblett P. (2013). Selenium levels in patients with different sources of sepsis. Crit. Care.

[74] Hughes D.J., Duarte-Salles T., Hybsier S., Trichopoulou A., Stepien M., Aleksandrova K., Overvad K., Tjønneland A., Olsen A., Affret A. (2016). Prediagnostic selenium Status and Hepatobiliary Cancer Risk in the European Prospective Investigation into Cancer and Nutrition Cohort. Am. J. Clin. Nutr..

[75] Chan S.-Y., Hsu C.-P., Ou Yang C.-H., Wang C.-C., Wu Y.-T., Fu C.-Y., Hsieh C.-H., Cheng C.-T., Lin W.-C., Huang J.-F. (2022). The Impact on the Clinical Prognosis of Low Serum selenium Level in Patients with Severe Trauma: Systematic Review and Meta-Analysis. Nutrients.

[76] Wang Q., Zennadi R. (2020). Oxidative Stress and Thrombosis during Aging: The Roles of Oxidative Stress in RBCs in Venous Thrombosis. Int. J. Mol. Sci..

[77] Hu X.F., Stranges S., Chan L.H.M. (2019). Circulating selenium Concentration Is Inversely Associated With the Prevalence of Stroke: Results From the Canadian Health Measures Survey and the National Health and Nutrition Examination Survey. J. Am. Heart Assoc..

[78] Hu X.F., Sharin T., Chan H.M. (2017). Dietary and Blood selenium Are Inversely Associated with the Prevalence of Stroke among Inuit in Canada. J. Trace Elem. Med. Biol. Organ Soc. Miner. Trace Elem. J. Trace Elem. Med. Biol..

[79] Wen Y., Huang S., Zhang Y., Zhang H., Zhou L., Li D., Xie C., Lv Z., Guo Y., Ke Y. (2019). Associations of Multiple Plasma Metals with the Risk of Ischemic Stroke: A Case-Control Study. Environ. Int..

[80] Hu H., Bi C., Lin T., Liu L., Song Y., Wang B., Wang P., Zhou Z., Fang C., Ma H. (2021). Sex Difference in the Association between Plasma selenium and First Stroke: A Community-Based Nested Case-Control Study. Biol. Sex Differ..

[81] Ding J., Zhang Y. (2022). Relationship between the Circulating selenium Level and Stroke: A Meta-Analysis of Observational Studies. J. Am. Nutr. Assoc..

[82] Mirończuk A., Kapica-Topczewska K., Socha K., Soroczyńska J., Jamiołkowski J., Kułakowska A., Kochanowicz J. (2021). selenium, Copper, Zinc Concentrations and Cu/Zn, Cu/Se Molar Ratios in the Serum of Patients with Acute Ischemic Stroke in Northeastern Poland-A New Insight into Stroke Pathophysiology. Nutrients.

[83] Fang H., Liu W., Zhang L., Pei L., Gao Y., Zhao L., Zhang R., Yang J., Song B., Xu Y. (2022). A Bidirectional Mendelian Randomization Study of selenium Levels and Ischemic Stroke. Front. Genet..

[84] Wei W.-Q., Abnet C.C., Qiao Y.-L., Dawsey S.M., Dong Z.-W., Sun X.-D., Fan J.-H., Gunter E.W., Taylor P.R., Mark S.D. (2004). Prospective Study of Serum selenium Concentrations and Esophageal and Gastric Cardia Cancer, Heart Disease, Stroke, and Total Death. Am. J. Clin. Nutr..

[85] Merrill P.D., Ampah S.B., He K., Rembert N.J., Brockman J., Kleindorfer D., McClure L.A. (2017). Association between trace elements in the environment and stroke risk: The reasons for geographic and racial differences in stroke (REGARDS) study. J. Trace Elem. Med. Biol. Organ Soc. Miner. Trace Elem..

[86] Wu Q., Sun X., Chen Q., Zhang X., Zhu Y. (2021). Genetically Predicted selenium Is Negatively Associated with Serum TC, LDL-C and Positively Associated with HbA1C Levels. J. Trace Elem. Med. Biol. Organ Soc. Miner. Trace Elem. J. Trace Elem. Med. Biol..

[87] Rayman M.P., Stranges S. (2013). Epidemiology of selenium and Type 2 Diabetes: Can We Make Sense of It?. Free Radic. Biol. Med..

[88] Yang J., Chen E., Choi C., Chan K., Yang Q., Rana J., Yang B., Huang C., Yang A., Lo K. (2022). Cross-Sectional Association of Blood selenium with Glycemic Biomarkers among U.S. Adults with Normoglycemia in the National Health and Nutrition Examination Survey 2013-2016. Nutrients.

[89] Sharifi-Razavi A., Karimi N., Jafarpour H. (2022). Evaluation of selenium Supplementation in Acute Ischemic Stroke Outcome: An Outcome Assessor Blind, Randomized, Placebo-Controlled, Feasibility Study. Neurol. India.

[90] Ramezani M., Simani L., Abedi S., Pakdaman H. (2021). Is selenium Supplementation Beneficial in Acute Ischemic Stroke?. Neurologist.

[91] Elkind M.S.V., Boehme A.K., Smith C.J., Meisel A., Buckwalter M.S. (2020). Infection as a Stroke Risk Factor and Determinant of Outcome After Stroke. Stroke.

[92] Antoniak S. (2018). The Coagulation System in Host Defense. Res. Pract. Thromb. Haemost..

[93] Zhao Y., Yang M., Mao Z., Yuan R., Wang L., Hu X., Zhou F., Kang H. (2019). The Clinical Outcomes of selenium Supplementation on Critically Ill Patients. Medicine.

[94] Leong P.K., Wong H.S., Chen J., Chan W.M., Leung H.Y., Ko K.M. (2016). Differential Action between Schisandrin A and Schisandrin B in Eliciting an Anti-Inflammatory Action: The Depletion of Reduced Glutathione and the Induction of an Antioxidant Response. PLoS ONE.

[95] Ishibashi N., Prokopenko O., Reuhl K.R., Mirochnitchenko O. (2002). Inflammatory Response and Glutathione Peroxidase in a Model of Stroke. J. Immunol..

[96] Tamburrano S., Rhodes S., Mosneag I.-E., Roberts L., Hurry M.E.D., Grainger J.R., Shaw T.N., Smith C.J., Allan S.M. (2022). Do Concentration or Activity of Selenoproteins Change in Acute Stroke Patients? A Systematic Review and Meta-Analyses. Cerebrovasc. Dis. Basel Switz..

[97] Taylor E.W., Ruzicka J.A., Premadasa L., Zhao L. (2016). Cellular Selenoprotein MRNA Tethering via Antisense Interactions with Ebola and HIV-1 MRNAs May Impact Host selenium Biochemistry. Curr. Top. Med. Chem..

[98] Premadasa L.S., Dailey G.P., Ruzicka J.A., Taylor E.W. (2021). Selenium-Dependent Read Through of the Conserved 3′-Terminal UGA Stop Codon of HIV-1 nef. Am. J. Biopharmacy Pharm. Sci..

[99] Gupta M., Copeland P.R. (2007). Functional analysis of the interplay between translation termination, selenocysteine codon context, and selenocysteine insertion sequence-binding protein 2. J. Biol. Chem..

[100] Vindry C., Guillin O., Mangeot P.E., Ohlmann T., Chavatte L. (2019). A Versatile Strategy to Reduce UGA-Selenocysteine Recoding Efficiency of the Ribosome Using CRISPR-Cas9-Viral-Like-Particles Targeting Selenocysteine-TRNA[Ser]Sec Gene. Cells.

[101] Vavougios G.D., Zarogiannis S.G., Krogfelt K.A., Stamoulis G., Gourgoulianis K.I. (2020). Epigenetic Regulation of Apoptosis via the PARK7 Interactome in Peripheral Blood Mononuclear Cells Donated by Tuberculosis Patients vs. Healthy Controls and the Response to Treatment: A Systems Biology Approach. Tuberc. Edinb. Scotl..

[102] Schwarz M., Lossow K., Schirl K., Hackler J., Renko K., Kopp J.F., Schwerdtle T., Schomburg L., Kipp A.P. (2020). Copper Interferes with Selenoprotein Synthesis and Activity. Redox Biol..

[103] Ravoori S., Srinivasan C., Pereg D., Robertson L.W., Ayotte P., Gupta R.C. (2010). Protective Effects of selenium against DNA Adduct Formation in Inuit Environmentally Exposed to PCBs. Environ. Int..

[104] Lai I.K., Chai Y., Simmons D., Watson W.H., Tan R., Haschek W.M., Wang K., Wang B., Ludewig G., Robertson L.W. (2011). Dietary selenium as a Modulator of PCB 126–Induced Hepatotoxicity in Male Sprague-Dawley Rats. Toxicol. Sci..

[105] Sugiura Y., Tamai Y., Tanaka H. (1978). selenium Protection against Mercury Toxicity: High Binding Affinity of Methylmercury by selenium-Containing Ligands in Comparison with Sulfur-Containing Ligands. Bioinorg. Chem..

[106] Limaye A., Yu R.-C., Chou C.-C., Liu J.-R., Cheng K.-C. (2018). Protective and Detoxifying Effects Conferred by Dietary selenium and Curcumin against AFB1-Mediated Toxicity in Livestock: A Review. Toxins.

[107] Cai H., Harrison D.G. (2000). Endothelial Dysfunction in Cardiovascular Diseases: The Role of Oxidant Stress. Circ. Res..

[108] Ivanov A.V., Bartosch B., Isaguliants M.G. (2017). Oxidative Stress in Infection and Consequent Disease. Oxid. Med. Cell. Longev..

[109] Ansari M.A., Ahmad A.S., Ahmad M., Salim S., Yousuf S., Ishrat T., Islam F. (2004). selenium Protects Cerebral Ischemia in Rat Brain Mitochondria. Biol. Trace Elem. Res..

[110] Zimmermann C., Winnefeld K., Streck S., Roskos M., Haberl R.L. (2004). Antioxidant Status in Acute Stroke Patients and Patients at Stroke Risk. Eur. Neurol..

[111] Angelova E.A., Atanassova P.A., Chalakova N.T., Dimitrov B.D. (2008). Associations between Serum selenium and Total Plasma Homocysteine during the Acute Phase of Ischaemic Stroke. Eur. Neurol..

[112] Song J., Park J., Oh Y., Lee J.E. (2015). Glutathione Suppresses Cerebral Infarct Volume and Cell Death after Ischemic Injury: Involvement of FOXO3 Inactivation and Bcl2 Expression. Oxid. Med. Cell. Longev..

[113] Morris D., Khurasany M., Nguyen T., Kim J., Guilford F., Mehta R., Gray D., Saviola B., Venketaraman V. (2013). Glutathione and Infection. Biochim. Biophys. Acta.

[114] Guerra C., Morris D., Sipin A., Kung S., Franklin M., Gray D., Tanzil M., Guilford F., Khasawneh F.T., Venketaraman V. (2011). Glutathione and Adaptive Immune Responses against Mycobacterium Tuberculosis Infection in Healthy and HIV Infected Individuals. PLoS ONE.

[115] Bizerea-Moga T.O., Pitulice L., Bizerea-Spiridon O., Moga T.V. (2021). Evaluation of Serum selenium Status by Age and Gender: A Retrospective Observational Cohort Study in Western Romania. Nutrients.

[116] Muntau A.C., Streiter M., Kappler M., Röschinger W., Schmid I., Rehnert A., Schramel P., Roscher A.A. (2002). Age-Related Reference Values for Serum selenium Concentrations in Infants and Children. Clin. Chem..

[117] Alehagen U., Johansson P., Björnstedt M., Rosén A., Post C., Aaseth J. (2016). Relatively High Mortality Risk in Elderly Swedish Subjects with Low selenium Status. Eur. J. Clin. Nutr..

[118] González S., Huerta J.M., Fernández S., Patterson E.M., Lasheras C. (2006). Food Intake and Serum selenium Concentration in Elderly People. Ann. Nutr. Metab..

[119] Zhou X., Smith A.M., Failla M.L., Hill K.E., Yu Z. (2012). Estrogen Status Alters Tissue Distribution and Metabolism of selenium in Female Rats. J. Nutr. Biochem..

[120] Griffiths N.M., Stewart R.D., Robinson M.F. (1976). The Metabolism of [75Se]Selenomethionine in Four Women. Br. J. Nutr..

[121] Janghorbani M., Christensen M.J., Nahapetian A., Young V.R. (1982). selenium Metabolism in Healthy Adults: Quantitative Aspects Using the Stable Isotope ^74^SeO_3_^2−^. Am. J. Clin. Nutr..

[122] Bratakos M.S., Zafiropoulos T.F., Siskos P.A., Ioannou P.V. (1988). selenium Losses on Cooking Greek Foods. Int. J. Food Sci. Technol..

[123] Li A., Zhou Q., Mei Y., Zhao J., Zhao M., Xu J., Ge X., Xu Q. (2022). Novel Strategies for Assessing Associations Between selenium Biomarkers and Cardiometabolic Risk Factors: Concentration, Visit-to-Visit Variability, or Individual Mean? Evidence from a Repeated-Measures Study of Older Adults With High selenium. Front. Nutr..

[124] Elkind M.S.V., Carty C.L., O’Meara E.S., Lumley T., Lefkowitz D., Kronmal R.A., Longstreth W.T. (2011). Hospitalization for Infection and Risk of Acute Ischemic Stroke: The Cardiovascular Health Study. Stroke.

[125] Cowan L.T., Alonso A., Pankow J.S., Folsom A.R., Rosamond W.D., Gottesman R.F., Lakshminarayan K. (2016). Hospitalized Infection as a Trigger for Acute Ischemic Stroke: The Atherosclerosis Risk in Communities Study. Stroke.

[126] Smeeth L., Thomas S.L., Hall A.J., Hubbard R., Farrington P., Vallance P. (2004). Risk of myocardial infarction and stroke after acute infection or vaccination. N. Engl. J. Med..

[127] Sebastian S., Stein L.K., Dhamoon M.S. (2019). Infection as a Stroke Trigger. Stroke.

[128] Boehme A.K., Ranawat P., Luna J., Kamel H., Elkind M.S.V. (2017). Risk of Acute Stroke After Hospitalization for Sepsis. Stroke.

[129] Shao I.Y., Elkind M.S.V., Boehme A.K. (2019). Risk Factors for Stroke in Patients With Sepsis and Bloodstream Infections. Stroke.

[130] Miller E.C., Elkind M.S.V. (2016). Infection and Stroke: An Update on Recent Progress. Curr. Neurol. Neurosci. Rep..

[131] Harms H., Halle E., Meisel A. (2011). Post-Stroke Infections—Diagnosis, Prediction, Prevention and Treatment to Improve Patient Outcomes. Eur. Neurol. Rev..

[132] Yuan Y., Xiao Y., Feng W., Liu Y., Yu Y., Zhou L., Qiu G., Wang H., Liu B., Liu K. (2017). Plasma Metal Concentrations and Incident Coronary Heart Disease in Chinese Adults: The Dongfeng-Tongji Cohort. Environ. Health Perspect..

[133] Salonen J., Alfthan G., Huttunen J., Pikkarainen J., Puska P. (1982). Association between cardiovascular death and myocardial infarction and serum selenium in matched-pair longitudinal study. Lancet.

[134] Blankenberg S., Rupprecht H.J., Bickel C., Torzewski M., Hafner G., Tiret L., Smieja M., Cambien F., Meyer J., Lackner K.J. (2003). Glutathione Peroxidase 1 Activity and Cardiovascular Events in Patients with Coronary Artery Disease. N. Engl. J. Med..

[135] Laclaustra M., Navas-Acien A., Stranges S., Ordovas J.M., Guallar E. (2009). Serum selenium Concentrations and Hypertension in the US Population. Circ. Cardiovasc. Qual. Outcomes.

[136] Bodnar M., Szczyglowska M., Konieczka P., Namiesnik J. (2016). Methods of selenium Supplementation: Bioavailability and Determination of selenium Compounds. Crit. Rev. Food Sci. Nutr..

[137] Bhaskar A., Munshi M., Khan S.Z., Fatima S., Arya R., Jameel S., Singh A. (2015). Measuring Glutathione Redox Potential of HIV-1-Infected Macrophages. J. Biol. Chem..

[138] Turck N., Robin X., Walter N., Fouda C., Hainard A., Sztajzel R., Wagner G., Hochstrasser D.F., Montaner J., Burkhard P.R. (2012). Blood Glutathione S-Transferase-π as a Time Indicator of Stroke Onset. PLoS ONE.

